# Adoptive Immunotherapy for Hematological Malignancies Using T Cells Gene-Modified to Express Tumor Antigen-Specific Receptors

**DOI:** 10.3390/ph7121049

**Published:** 2014-12-15

**Authors:** Hiroshi Fujiwara

**Affiliations:** Department of Hematology, Clinical Immunology and Infectious Disease, Graduate School of Medicine, Ehime University, Toon, Ehime 791-0295, Japan; E-Mail: yunarief@m.ehime-u.ac.jp

**Keywords:** adoptive immunotherapy, gene-modified T cell, T-cell receptor, chimeric antigen receptor, hematological malignancy

## Abstract

Accumulating clinical evidence suggests that adoptive T-cell immunotherapy could be a promising option for control of cancer; evident examples include the graft-*vs*-leukemia effect mediated by donor lymphocyte infusion (DLI) and therapeutic infusion of *ex vivo-*expanded tumor-infiltrating lymphocytes (TIL) for melanoma. Currently, along with advances in synthetic immunology, gene-modified T cells retargeted to defined tumor antigens have been introduced as “cellular drugs”. As the functional properties of the adoptive immune response mediated by T lymphocytes are decisively regulated by their T-cell receptors (TCRs), transfer of genes encoding target antigen-specific receptors should enable polyclonal T cells to be uniformly redirected toward cancer cells. Clinically, anticancer adoptive immunotherapy using genetically engineered T cells has an impressive track record. Notable examples include the dramatic benefit of *chimeric antigen receptor* (*CAR*) gene-modified T cells redirected towards CD19 in patients with B-cell malignancy, and the encouraging results obtained with *TCR* gene-modified T cells redirected towards NY-ESO-1, a cancer-testis antigen, in patients with advanced melanoma and synovial cell sarcoma. This article overviews the current status of this treatment option, and discusses challenging issues that still restrain the full effectiveness of this strategy, especially in the context of hematological malignancy.

## 1. Introduction

Clinically available adoptive immunotherapies employing non-gene-modified T cells have successfully demonstrated the potential for cure of certain cancers and viral infections. Examples include the graft-*vs*-leukemia (GVL) effect mediated by donor lymphocyte infusion (DLI) for treatment of relapsed leukemia and persistent reactivation of latent viruses after allogeneic stem cell transplantation (allo-HSCT) [1,2], and therapeutic infusion of *ex vivo*-expanded tumor-infiltrating lymphocytes (TIL) in combination with lymphodepletion for treatment of advanced melanoma [3,4]. This lymphodepleting preconditioning augments the antitumor response by eliminating regulatory T cells, by reducing total T cells which are a “sink” for cytokines including IL-7 and IL-15 playing important roles in “homeostatic expansion”, and by augmenting the activation and availability of antigen-presenting cells [5,6,7]. However, both DLI and TIL therapy have fundamental drawbacks; the former is usually associated with a potentially life-threatening form of allo-immunity-mediated damage to normal tissue, graft-*vs*-host disease (GVHD) [8], whereas the latter requires time-consuming and labor-intensive procedures for preparation of therapeutic cells, and the results are sometimes unsuccessful [9]. Separation of the GVL effect from GVHD still remains the main challenge in allo-HSCT, and to achieve this, attempts have been made to discover effective immunogenic antigens that can be selectively or abundantly expressed in leukemia cells, but not in normal tissues [10].

In order to overcome these drawbacks, the concept of genetically modifying naturally occurring effector T cells so that they become specifically responsive to therapeutic targets has been developed as an alternative approach. At present, gene-modified T cell-based adoptive immunotherapy is still evolving in order to achieve durable suppression of cancer progression, which is prerequisite for cure. Preclinical and clinical studies using adoptive T-cell transfer have revealed that cytotoxic CD8^+^ T cells (CTLs) are primarily advantageous in comparison with other cytolytic cells, such as NK cells, because they are capable of specifically targeting tumor cells through recognition of differentially expressed cell surface antigens, and also clonal proliferation after target recognition, resulting in durable suppression of cancer cells [11]. The long life span of clonal T cells also seems attractive from the viewpoint of not only therapeutics, but also immuno-prophylaxis to durably reduce the incidence of disease in patients with a high risk of cancer [12]. As is the case with TIL therapy, the anticancer efficacy of infused gene-modified T cells can also be enhanced using lymphodepletion [13]. Furthermore, T cells are able to tolerate gene modification using viral vectors and transposons [14]. This makes T cells suitable for transduction with target antigen-specific receptor genes, making it possible to uniformly redirect large numbers of polyclonal T cells towards cancer cells on a large scale. In the context of these technological advances, adoptive immunotherapy using genetically retargeted T cells has become a realistic treatment option for cancer and refractory viral infection [15]. The present review summarizes the current status of anticancer adoptive immunotherapy using gene-modified T cells, and discusses the various issues that still blunt the full effectiveness of this strategy, particularly for hematological malignancy.

## 2. Gene-Modified T Cells Redirected Towards Therapeutic Targets

Through the development of anti-melanoma TIL therapy [3,4] employing *ex vivo*-expanded T cells with guaranteed tumor reactivity and tumor trafficking activity, several requisite conditions for a favorable clinical outcome of adoptive T-cell immunotherapy have become apparent: (i) the choice of an optimal target antigen; (ii) to the extent possible guaranteed uniformity of the quantity and quality of the therapeutic T cells administered; and (iii) tumor trafficking and *in vivo* persistence of the infused T cells [16]. Ideal effector T cells should bear a single T-cell receptor with high affinity optimized to an appropriately selected target antigen, and should be capable of actively proliferating upon recognition of the target cells, durably persisting thereafter *in vivo*. In addition, such effector T cells need to be prepared in sufficient numbers in a timely manner.

**Figure 1:**
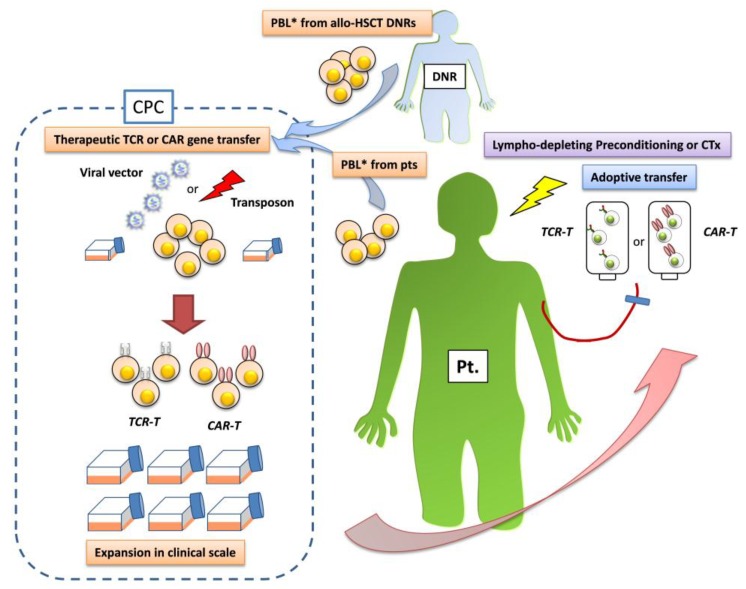
Flow chart of antileukemia adoptive immunotherapy using gene-modified T cells (modified from [18]).

At present, retro- or lentiviral, or electroporational transfer of chimeric antigen receptors (CARs) whose target recognition is dependent on a single-chain variable region domain of a monoclonal antibody (scFv) or that of a T-cell receptor (TCR) is employed for stable production of sufficient numbers of therapeutic T cells (CAR-T cells or TCR-T cells, respectively) uniformly retaining specificity for their defined targets, generally in 2-week culture [15]. Accordingly, the concept that, in the near future, therapeutic gene-modified T cells may be employed an “off-the-shelf” cellular drug and produced on an industrial scale is becoming accepted worldwide, encouraged by proactive measures by the US Food and Drug Administration in balancing the benefits and risks to subjects in clinical trials [17]. Figure 1 shows a flow chart of adoptive immunotherapy using gene-modified T cells.

PBL harvested from a patient with leukemia in CR or a HLA-identical allo-HSCT donor using leukapheresis are subjected to gene modification with a relevant TCR or CAR. Subsequently, TCR-T cells or CAR-T cells are expanded on a clinical scale at a cell processing center. A sufficient number of TCR-T or CAR-T cells are then infused back into the same patient, with or without chemo-radiotherapeutic preconditioning. PBL: peripheral blood lymphocytes, CR: complete remission, TCR: T cell receptor, CAR: chimeric antigen receptor, *TCR-T*: *T cell receptor* gene-transduced T cells, *CAR-T* : *chimeric antigen receptor* gene-transduced T cells, CPC: Cell processing center, Pt: patient, DNR: donor, CTx: chemotherapy.

**Figure 2:**
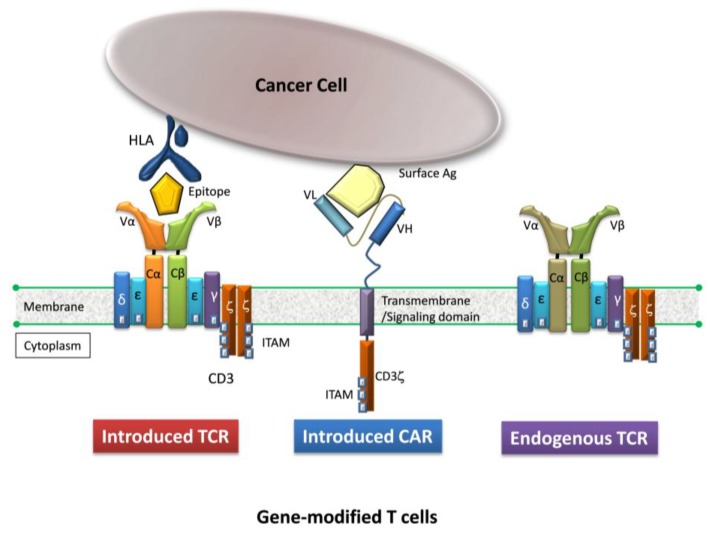
A T cell gene-modified to express a TCR or CAR (modified from [18]).

Given that allo-HSCT, being the most successful type of adoptive therapy, requires timely acquisition of hematopoietic stem cells from the more appropriate donor, the gene-modification using autologous lymphocytes from cancer-bearing patients, not requiring donors, can provide the higher clinical versatility. On the other hand, gene-modification of allogeneic T cells from an allo-HSCT donor for DLI is greatly advantageous to employ chemo-naïve healthy T cells. In Figure 2, the basic structure of TCR-T and CAR-T cells is shown [18].

The introduced TCR (left) or CAR (right) construct in a gene-modified T cell is illustrated. The TCR recognizes the epitope derived from cytoplasmic antigens in the context of the particular HLA (left), whereas the CAR recognizes the cell surface antigen on the basis of the recognition machinery of a monoclonal antibody, independently of HLA (right). TCR: T cell receptor, CAR: chimeric antigen receptor, HLA: human leukocyte antigen, Vα: variable region of the TCR α-chain, Vβ: variable region of the TCR β-chain, Cα: constant region of the TCR α-chain, Cβ: constant region of the TCR β-chain, ε, γ, δ, ζ: each subunit of the CD3 molecule, Ag: antigen, VL: variable region of the immunoglobulin light chain, VH: variable region of the immunoglobulin heavy chain, ITAM: immunoreceptor tyrosine-based activation motif.

The TCR-T cell, but not the CAR-T cell, requires a specific HLA molecule for recognition of the target antigen which is processed within the cytoplasm and presented on the surface of cancer cells, *i.e.*, HLA restriction. In other words, limited individuals who inherently have particular HLA molecules are eligible for this treatment option. Compared to CAR-T cells, such HLA restriction limits clinical versatility for TCR-T cells to some extent, however TCR-T cells, but not CAR-T cells, have the ability to recognize intracellular proteins, providing a broader array of target tumor-associated antigens [19].

The therapeutic quality of gene-modified TCR-T cells is primarily dependent on their avidity. Because of thymic selection during the development of T cells and the fact that except for a few mutated proteins encoded by cancer-causing genetic mutations (driver mutations), a large proportion of tumor antigens are self-antigens, and circulating T cells have already been massively exposed to tumor antigens in cancer-bearing patients, naturally occurring TCRs expressed on circulating T cells generally have low affinity for self-antigens (Kd range 1–100 μM) [20]. This makes such circulating T cells less responsive to autologous cancer cells, because generally cancer cells tend to express small amounts of epitope/HLA complexes on their surface [21]. Therefore, to create higher avidity, several strategies have been developed with the aim of increasing the affinity of the relevant TCR beyond that expressed by naturally occurring T cells in response to an identical epitope. These strategies include the use of selected TCRs from immunized human HLA transgenic mice with relevant epitopes and insertion of targeted mutation in complementary-determining region 2 or 3 (CDR2 or 3) in the variable regions of the TCR α/β chains that interact with the HLA/epitope complex [22,23,24,25,26]. For example, a mutation encodes Alanine to Threonine amino acid substitution in CDR3 region of the TCR-α chain of MAGE-A3-specific TCR at position 118 is inserted [25]. Such affinity-increased TCRs have already been employed in clinical trials [25,26,27,28,29]. Those affinity-increased TCRs that were included figure in Table 1 and are highlighted with *. Six of 20 patients with advanced melanoma treated with gene-modified T cells using a HLA-A*0201-restricted melan-A protein and melanoma antigen recognized by T cells 1 (MART-1)-specific TCR achieved partial remission (PR), those were suffered from dermatitis and uveitis (grade2) [27]. One of three patients with advanced colorectal carcinoma treated with gene-modified T cells using a HLA-A*0201-restricted carcinoenbryonic antigen (CEA)-specific TCR showed PR, but three of three patients contracted treatment-required colitis (grade 3) [28]. Two of 11 patients with advanced melanoma HLA-A*0201-restricted NY-ESO-1-specific TCR demonstrated complete remission (CR) and three of 11 achieved PR. Additionally four of six patients with metastatic synovial cell carcinoma treated with the same NY-ESO-1-specific TCR achieved PR. None of the patients demonstrated any adverse events [29]. Five of nine patients (seven patients with advanced melanoma, one metastatic synovial cell carcinoma and one advanced esophageal carcinoma) treated with gene-modified T cells using a HLA-A*0201-restricted melanoma antigen family A3 (MAGE-A3)-specific TCR demonstrated tumor regression, but two patients died of severe central nervous system damage [25]. Two of two patients (one advanced melanoma and one advanced myeloma) treated with gene-modified T cells using HLA-A*01-restricted TCR died of severe cardiac dysfunction shortly after T-cell infusion [26]. TCR-T-associated adverse events are discussed later.

On the other hand, it is widely accepted that introduction of a therapeutic TCR can create an unwanted α/β heterodimer between the introduced and endogenous TCR α/β chains (mispairing). Mispairing can directly reduce the cell-surface expression of intended TCR α/β pairing, resulting in low avidity. In turn, cancer cells in daily practice show low cell surface expression of the relevant epitope/HLA complex, accordingly gene-modified T cells expressing mispaired TCRs seem apt to fail to recognize cancer cells [30,31].

Furthermore, the potential risk of severe autoimmune disease caused by newly generated mispaired TCR with unpredictable auto-responsiveness has been highlighted in a mouse model [32], although so far no such adverse event has been observed in clinical trials. To circumvent mispaired TCR formation, knockdown or knockout of endogenous *TCR*-α/β gene expression has recently been introduced using *siRNA* technology (*siTCR* vector) [33,34] or *zinc-finger nuclease* (*ZFN*) technology [35,36].

**Table 1:** Results from clinical trials using TCR-T cells.

Taregt Ag. of TCR	Cell Dose (× 10^9^)	Target Disease Pt.No. (n)	Preconditioning	AEs (Grade)	Clinical Responses	Ref.
MART-1	1.0–86	melanoma	Cy + Flud	No	PR 2/17	[37]
		(n = 17)				
MART-1*	1.5–107	melanoma (20)	Cy + Flud	skin, eye (G2)	PR 6/20	[27]
gp100**	1.8–110	melanoma (16)		ear (G3)	CR 1/16,PR 2/16	
		(n = 36)				
p53**/gp100**	0.5–27.7	breast ca. (4)	Cy + Flud	N/A	PR 1/9	[38]
		melanoma (2)				
		esoph.ca. (1)				
		others (2)				
CEA*	0.2–0.4	colorectal ca.	Cy + Flud	colitis (G3)	PR 1/3	[28]
		(n = 3)			CEA decreased 3/3	
NY-ESO-1*	1.6–130	melanoma (11)	Cy + Flud	No	CR 2/11, PR 3/11	[29]
		synovial cell ca.(6)			PR 4/6	
		(n = 17)				
MAGE-A3*	29–79	melanoma (7)	Cy + Flud	mental disturbance (G4)	Tumor regression	[25]
		synovial cell ca.(1)		2/3 died of necrotizing	5/9	
		esoph.ca. (1)		leukoencephalopathy		
		(n = 9)		(on-target AE)		
MAGE-A3*	5.3 & 2.4	melonoma (1)	Cy	2/2 died of cardiogenic	NE	[26]
		myeloma (1)	melphalan + autoSCT	shock		
		(n = 2)		(off-target AE)		

*Abbreviations*: Ag: antigen; AE: adverse events; Ref.: reference; Cy: cyclophosphamide; Flud: fludarabine; PR: partial response; CR: complete remission by response evaluation criteria in solid tumors (RECIST) *: affinity-increased TCR; **: mouse-derived high-avidity TCR; breast ca.: breast cancer; esoph.ca.: esophageal cancer; synovial cell ca.: synovial cell carcinoma; N/A; not available; N/E: not evaluable; autoSCT: autologous stem cell transplant.

siTCR vector simultaneously transduces therapeutic *TCR*-α/β genes and *siRNA* for constant regions of endogenous *TCR*-α/β genes for the suppression in gene-modified T cells. Therapeutic *TCR*-α/β genes were codon-optimized to be resistant to those *siRNAs*. Accordingly, mispairing between introduced and endogenous *TCR*-α/β genes can be reduced. In cooperation with Takara Bio Inc. and Mie University, we have originally developed the *siTCR* vector system, and recently launched a clinical trial using a therapeutic *siTCR* vector targeting a leukemia antigen, Wilms Tumor 1 (WT1-*siTCR* vector) [39], for patients with acute myelogenous leukemia (AML) and myelodysplastic syndrome (MDS) (UMIN 00001159). Silencing of the *CC chemokine 5* (*CCR5*) gene in CD4^+^ T cells using *ZFN* technology has also been used in a clinical trial for patients with human immunodeficiency virus (HIV) infection (NCT00842634, ClinicalTrials.gov.Identifier), and shown to be clinically safe [40]. However, at the time of writing, no clinical trial of anticancer adoptive immunotherapy using *ZFN* –manipulated T cells had been reported.

As shown in Figure 2, the CAR construct is composed of an extracellular antigen-binding domain, a transmembrane domain, and a cytoplasmic signaling domain [41]. First-generation CAR constructs solely containing either CD3-ζ or FcR-γ as a cytoplasmic signaling molecule [42,43] were clinically unsuccessful [44]. Limitations in the proliferative response following recognition of tumor cells, and in persistence and homing of first-generation CAR-T cells to local tumor cells *in vivo* have been largely surmounted by second-generation CAR constructs containing co-stimulatory signaling molecules such as CD28 [45], 4-1BB [46], OX40 [47] and ICOS (inducible T-cell co-stimulator; CD278) [48], resulting in upregulation of anti-apoptotic factors and increased secretion of cytokines upon antigen recognition [49,50]. Consequently, clinical outcomes resulting from the use of second-generation CAR-T cells have been dramatically improved, particularly for B-cell malignancy [51,52,53,54,55,56]. For further upregulation of antitumor functionality mediated by CAR-T cells, third-generation CAR constructs are currently being investigated [41]. On the other hand, a better understanding of the extracellular domain is still emerging [57]. In addition, as was the case with TCR-T cells [4], preconditioning for lymphodepletion prior to transfer of CAR-T cells also seems to affect the functionality of these effector cells [58].

## 3. Lessons from Clinical Trials Using T Cells Gene-Modified by Tumor Antigen-Specific *Receptor* Gene Transfer

Results from major clinical trials using TCR-T cells and CAR-T cells are summarized separately in Table 1 and Table 2. Promising outcomes have been achieved in clinical trials using CD19-specific CAR constructs for patients with refractory chronic lymphocytic leukemia [51,52,54,59], B-cell [53,55,59,60], and acute lymphoblastic leukemia [54,61]. Interestingly, more impressive clinical benefits seemed to be achieved using the CD19-CAR construct containing 4.1BB in the signaling motif [51,52,61] than with the use of CD28 [53,54], although the mechanism underlying this difference in antitumor efficacy is not fully understood. Even if the same CD19 is targeted, because of the differences in the cytoplasmic domain and the extracellular domain recognizing a different epitope of the same antigen with differing affinity for each CAR construct, the antitumor functionality mediated by CAR-T cells varies [62]. As to the importance of the extracellular domain, studies of the anti-CD22 CAR construct have revealed that the extent of antitumor efficacy mediated by each anti-CD22 CAR-T cell widely varies according to the individual extracellular domain that recognizes a different epitope, even when derived from the same CD22 molecule [63].

**Table 2:** Results from clinical trials using CAR-T cells.

Target Ag. of CAR	Cell Dose	Target disease Pt.No. (n)	Preconditioning	AEs (Grade)	Clinical Responses	Ref.
L1-cell adhesion	1 × 10^8^	neuroblastoma (6)	none	pancytopenia (G3)	PR 1/6	[64]
molecule/CAR*	Or 10^9^/m^2^			bacteremia, pneumonitis		
HER2/CAR**	1 × 10^1^°	colon cancer with	Cy + Flud	died of acute pulmonary	N/E	[65]
		lung/liver meta. (1)		failure		
	
GD2/CAR*	0.2–0.5–1	neuroblastoma (19)	none	no	CR 3/19, PR 1/19	[66]
EBV-CTL	× 10^8^					
CD19/CAR***	1.1 × 10^9^	CLL (3)	CTx for CLL	lymphopenia (G3)	CR	[51]
	5.8 × 10^8^			B cell aplasia	PR	
	1.4 × 10^7^				CR	
CD19/CAR^#^	1.0–11.1	CLL (8)	none for 3	hypotension (G3)	PR 1/8 in CLL	[53]
	× 10^9^	ALL (2)	Cy (1500 mg or 3000 mg)	1 died of shock, renal failure	B cell aplasia	
			for others	B cell aplasia		
CD19/CAR^#^	0.5–5.5	CLL (4)	Cy + Flud	hypotension (G3/4)	CR 1/4, PR 2/4 in CLL	[52]
	× 10^7^ /Kg	FL (4)		renal failure, infection	PR 3/4 in FL	
				B cell aplasia	B cell aplasia	
CD20/CAR**	4.4 × 10^9^ /m^2^	MCL (2)	Cy (1000 mg/m^2^ )	hypoxia (G3), fever (G2)	PR in FL	[67]
		FL (1)				
CD19/CAR^#^	1.0–11.1	B-ALL (5)	Cy (1500 mg or 3000 mg)	fever (G2)	CR 2/6 (no MRD)	[54]
	× 10^6^ /Kg					
CD19/CAR***	1.4–12	ALL (2)	CTx for ALL	CRS (G3-4)	CR 2/2	[55]
	× 10^6^ /Kg			B cell aplasia		
CD19/CAR *^#^*	3 × 10^6^ /kg	refractory B-ALL (16)	Cy (1500 mg or 3000 mg)	CRS (G3-4) (7/16)	CR 14/16	[56]
		ph+ (4/16)		neurologic complication (3/7)	molecular CR 10/14	
				respiratory ventilation (3/3)	transit to allo-HSCT	
					(7/14)	

*Abbreviations*: Ag.: antigen; AE: adverse events; Ref.: reference; Cy: cyclophosphamide; Flud: fludarabine; PR: partial response; CR: complete remission; CTx: chemotherapy; CLL: chronic lymphocytic leukemia; (B-)ALL: (B-cell) acute lymphoblastic leukemia; MCL: mantle cell lymphoma; FL: follicular cell lymphoma; HER2: human epitherial growth factor receptor 2; EBV: Epstein-Barr virus; CTL: cytotoxic T lymphocyte; *: first generation CAR: **: CD28,4.1BB bearing CAR; ***: 4.1BB bearing 2nd generation CAR; #: CD28 bearing 2nd generation CAR; N/A: not available; N/E: not evaluable: CRS: cytokine releasing syndrome; allo-HSCT: allogeneic hematopoietic stem cell transplantation.

Nonetheless, the contribution of high-performance 2nd and 3rd generation CAR constructs to the clinical success of anti-CD19 CAR-T cell therapy for B cell malignancy is largely attributable to the nature of CD19 itself: (i) CD19 is expressed not only by leukemia cells, but also by normal antigen-presenting B cells that can provide co-stimulatory signals to CAR-T cells upon contact, (ii) infused CAR-T cells are easily accessible to leukemia cells in the same physiological compartment (lympho-vascular system), and (iii) apart from unknown late-phase adverse events [68], adverse symptoms associated with B-cell depletion appear to be clinically manageable using intravenous administration of immunoglobulin, *i.e.*, B cells can be considered to be a relatively non-essential normal tissue. The clinical success of anti-CD19 CAR-T cell therapy again underlies the importance of choice of the optimal target antigen for gene-modified T cells.

On the other hand, with regard to TCR-T cell therapy, Rosenberg and colleagues have been playing a leading role, particularly in the treatment of advanced melanoma [27,37] and solid tumors [28]. They have recently reported impressive clinical outcomes with the use of a genetically mutated high-affinity TCR specific to NY-ESO-1 in the context of HLA-A*02:01, a representative cancer-testis antigen (CTA) that is overexpressed in various types of cancers, but negligibly expressed in almost all normal tissues except for testis [29]. Infusion of NY-ESO-1-specific TCR-T cells resulted in tumor regression in five of six patients with advanced melanoma, including two patients who achieved complete remission for longer than 1 year, and four of six patients with synovial cell carcinoma. There is a concern that a TCR expressing supra-physiologic affinity might have an increased risk to damage normal tissues that physiologically express the same target antigen even at a negligible level (on-target adverse effect), but not when NY-ESO-1 is targeted. Again, naturally occurring CTLs in the circulation of a cancer-bearing patient are less responsive to autologous cancer cells [21]. Of necessity, a technology for artificially upregulating the affinity of a relevant TCR to a level as high as that of a monoclonal antibody (Kd value < nM) has been developed. Accordingly, emerging on- and off-target adverse effects mediated by such artificial TCRs with supra-physiologic affinities have become a serious issue, and will be discussed later.

In the context of hematological malignancy, no clinical observations of TCR-T therapy have yet been reported. Currently, three clinical trials using TCR-T cells targeting WT1 are ongoing. The first is being conducted by our group in Japan using autologous gene-modified T cells with a naturally occurring HLA-A*24:02-restricted WT1-specific TCR using a *siTCR* vector without lymphodepletion for the treatment of AML/MDS (UMIN000011519). The second is being conducted by Greenberg and colleagues in the USA using allogeneic gene-modified T cells with a naturally occurring and selected high-affinity HLA-A*02:01-restricted WT1-specific TCR in combination with rhIL-2 administration, but without using lymphodepletion, for the treatment of relapsed or high-risk AML/MDS/CML after allo-HSCT (ClinicalTrials.gov. Identifier NCT01640301). This group has previously reported the results of a similar clinical trial using *ex vivo*-expanded allogeneic donor-derived CTL clones employing repetitive stimulation of the HLA-A*02:01-restricted WT1 epitope for treatment of relapsed or high-risk AML after allo-HSCT [69]. In the same study, infused allogeneic donor WT1-specific CTLs exerted an antileukemia effect without exacerbating GVHD, and those CTLs pre-cultured with a rhIL-21 expressing memory T-cell phenotype achieved a notably durable leukemia remission for longer than 30 months [69]. In the current clinical trial, gene-modified CD8^+^ T cells specific to the identical WT1 epitope in the context of HLA-A*02:01, in turn, are being employed. The third trial is being conducted by Morris and colleagues in the UK using autologous gene-modified T cells with a HLA-A*02:01-restricted WT1-specific TCR in combination with lymphodepletion for treatment of AML/CML (ClinicalTrials.gov. Identifier NCT01621724). Reports from these clinical trials are eagerly anticipated.

## 4. Adverse Effects of Gene-Modified T Cells

The adverse effects (AE)s caused by adoptively administered gene-modified T cells can largely be divided into: (i) on-target AE, which is damage due to the expression of target antigens in normal tissues recognized by the introduced CAR or TCR, and (ii) off-target AE, which is damaging to normal tissues that do not express the antigens targeted by the introduced CAR or TCR. Despite obvious tumor shrinkage, on-target AEs have been observed in a clinical trial using an avidity-increased HLA-A*0201-restricted TCR specific for the melanoma-associated antigens MART-1 and gp100, which are also expressed substantially in normal melanocytes and pigmented tissues, resulting in skin rash, hearing loss and uveitis [27]. When carcinoembryonic antigen (CEA), also constitutively expressed by normal colon epithelia, was targeted using T cells gene-modified to express a HLA-A*0201-restricted and CEA-specific high-avidity murine TCR, severe inflammatory colitis was observed in all of three colon cancer patients who received this treatment [28]. There is no doubt that high-avidity therapeutic TCR-T cells without an increased incidence of treatment-related on-and off-target AEs is desirable to achieve a better anticancer effect [70]. It is anticipated that this dilemma would be overcome by selection of appropriate target antigens, for example CTA, as has been demonstrated successfully for T cells gene-modified using an affinity-enhanced TCR targeting NY-ESO-1 [29]. Quite recently, however, it has been reported that T cells gene-modified using engineered TCRs with supra-physiologic affinities (Kd values <nM) specific for MAGE-A3, one of the leading CTAs, have caused fatal damage to vital organs, including the central nervous system (CNS) [25] and heart [26]. It is interesting that CDR2 was genetically engineered in both TCRs. In the former study, the HLA-A*02:01-restricted MAGE-A3-specific TCR, having recognized MAGE-A3/9/12 family proteins, caused unexpected severe neurological toxicity due to cross-reactivity with an epitope on MAGE-A12 expressed in the white matter of the brain (on-target AE) [25]. On the other hand, the HLA-A*01-restricted MAGE-A3-specific TCR employed in the latter study caused fatal cardiac toxicity due to an unexpected cross-reaction with the epitope on the cardiac muscle protein titin, which happened to have an amino acid sequence analogous to the original target on MAGE-A3 (off-target AE) [26]. The most important lesson from these cases is that in the strict sense, a serious AE is not predictable, even if the chosen antigen is appropriate and intensive preclinical studies have been performed. Accordingly, the authors of the latter report have proposed a novel screening system using a relevant HLA-expressing vital organ cell panel generated from induced pluripotent stem cells (iPSCs) to prospectively detect any risk of serious self-reactivity mediated by T cells gene-modified to express TCRs with supra-physiologic affinity.

Anti-CD19 CAR-T cell therapy, currently building on its success, also has problems to be solved. Normal B-cell aplasia in patients treated with anti-CD19-CAR-T cells represents another on-target AE that has so far been manageable using immunoglobulin replacement [51,55], although this problem still remains an unaddressed in the long term, and may have an economic impact on healthcare. Once a B-cell malignancy has been eradicated, any remaining anti-CD19-CAR-T cells should be ablated in order to facilitate normal B-cell reconstitution. A fatal on-target AE has also been observed during CAR-T-cell therapy. This was a case of serious pulmonary failure ending in multi-organ failure due to a massive release of cytokine in a patient with metastatic colon cancer who received CAR-T cells specific to human epithelial growth factor 2 (Her2, CD340) in combination with lymphodepletion [65]. This Her2-specific CAR construct comprised CD28, 4-1BB and CD3ζ in the cytoplasmic signaling domain, *i.e.*, a “souped-up” 3rd-generation CAR construct. Although at a low level, Her2 was broadly expressed in pulmonary epithelial cells, and this was assumed to have caused the severe pulmonary injury as an on-target AE.

A braking system by which T cells are inherently equipped for excessive activation via TCR signaling, *i.e.*, activation-induced cell death (AICD), might also prevent overproduction of TCR-T cells [71]. On the other hand, because CAR-T cells can non-physiologically overcome barriers to proliferation and persistence, a serious off-target AE directed against normal tissues may be characterized by massive release of pro-inflammatory cytokines *in vivo* from long-lasting hyperactive CAR-T cells, *i.e.*, cytokine release syndrome (CRS). For example, transient cardiac dysfunction observed in patients who had received anti-CD19-CAR-T cells was not due to CD19 expression in heart tissue, and elevated serum levels of IFN-γ, TNF-α and IL-6 were observed [51,53]. In a recent clinical trial using anti-CD19-CAR-T cells, seven of 16 patients with chemotherapy-resistant B-ALL developed CRS including three cases of serious consciousness disturbance requiring ventilator support [56]. In the same report, the authors also highlighted the efficacy of an anti-IL-6 receptor mAb (tosilizumab) for treatment of severe CRS and the value of CRP for predicting its incidence [72]. Another off-target AE is tumor lysis syndrome (TLS) due to rapid and massive destruction of tumor cells, being characterized pathophysiologically by extremely elevated levels of serum phosphate, potassium and uric acid. These symptoms considerably overlap those of CRS [72]. In the clinical trial described above, the severity of CRS was positively correlated with the extent of the residual tumor cell burden upon infusion of anti-CD19-CAR-T cells [56]. Such CRS upon infusion of CAR-T cells was finally managed successfully by rearrangement of the CAR-T cell transfusion schedule, thus avoiding the risk of TLS [73]. Another occasionally life-threatening off-target AE is macrophage activation syndrome (MAS), characterized by systemic inflammatory symptoms and pancytopenia due to hemophagocytosis in the bone marrow, although its precise mechanism is still undetermined [56]. Another severe transient cardiac dysfunction as an off-target AE was experienced in a clinical trial using CAR-T cells specific for mesothelin. This event was due to anaphylaxis mediated by formation of a human anti-mouse antibody against the murine component of the CAR construct [74].

## 5. Attempts to Address Challenging Issues

**Figure 3:**
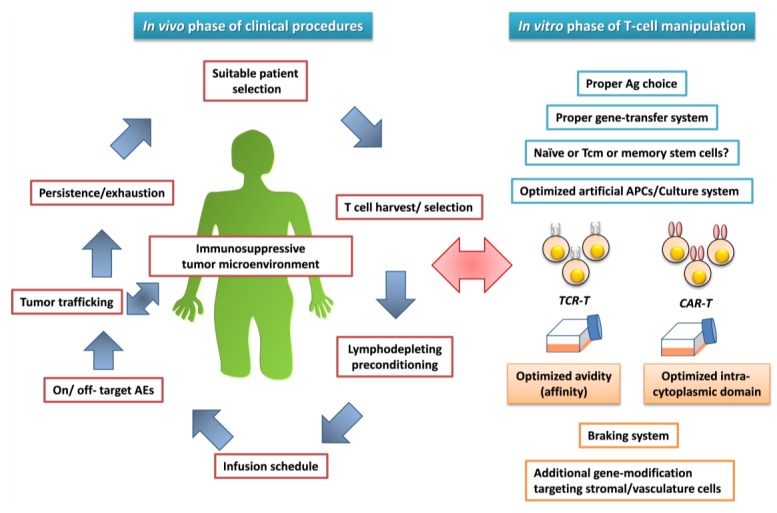
Unsolved issues for the establishment of successful antileukemia adoptive immunotherapy using gene-modified T cells.

The improved clinical outcomes resulting from the development of lymphodepletion [3,4] and the use of anti-CD19-CAR-T cells for B-cell malignancies [51,52,53,54,55,56,59,60,61] suggest that many of the problems associated with gene-modified T-cell-based anticancer adoptive immunotherapy have been solved. However, a number of unsolved issues remain to be addressed before this treatment option can be widely accepted in a clinical setting (Figure 3).

For the establishment of successful antileukemia adoptive immunotherapy using gene-modified T cells, various factors still remain unresolved in both the *in vitro* phase of T-cell manipulation (right) and the *in vivo* phase of clinical procedures (left). These factors mutually impact each other and eventually dictate the clinical outcome. Factors involved in the *in vitro* phase of T-cell manipulation will impact on the quality and quantity of the gene-modified T-cell product. On the other hand, factors associated with the clinical procedures would include how and when the T cells are harvested, how the gene-modified T cells are infused, *i.e.*, with or without lymphodepletion, how unwanted on- and off-target AEs can be avoided, how the gene-modified T cells accumulate in local tumor tissues, and how the infused T cells are able to remain durably viable and active thereafter *in vivo*. At the same time, it is also important to overcome the immunosuppressive tumor microenvironment in patients with leukemia. Details are discussed in the *Attempts to address challenging issues* section. Ag: antigen, DC: dendritic cells, APC: antigen-presenting cells, *TCR-T* : relevant *TCR* gene-modified T cells, *CAR-T* : relevant *CAR* gene-modified T cells, AE: adverse effect.

Above all, in order to avoid serious on-target AEs, the choice of an appropriate antigen is indisputably important. The ideal target antigen should be selectively expressed by cancer cells, but absent in normal tissues, and sufficiently immunogenic to generate T-cell responses, resulting in effective anticancer reactivity without damage to normal cells. Strictly, two types of antigen meet these requirements: (i) viral antigens in virus-induced tumors, and (ii) mutated antigens encoded by causative gene alterations in cancer cells. It is anticipated that CAR-T cells or TCR-T cells, which are harnessed to the defined viral epitopes, would be able to eliminate virus-transformed cancers. In addition, whole-exon sequencing of cancer cells has recently identified novel candidate mutations in melanoma [75]. In the context of leukemia, identification of such mutations in a leukemia stem cell may open a new door to immunotherapy in the near future [76].

In order to establish the more powerful gene-modified T cells against cancer, TCR-T cells bearing supra-physiologic affinities and CAR-T cells displaying unnaturally enhanced proliferation and persistence upon target recognition have been developed, and both resulting in overwhelming extension of functional range of naturally occurring T cells. Accordingly, serious on- or off-target AEs have inevitably emerged, and these AEs are difficult to predict using conventional methods. An iPS-derived vital-organ cell panel expressing common HLA haplotypes might provide a breakthrough in this respect [26].

On the other hand, a novel vector system that simultaneously encodes a suicide system has been developed in order to eliminate gene-modified T cells when they display unwanted toxicities mediated by infused gene-modified T cells, for example, *thymidine kinase* gene of herpes simplex virus [77]. Most recently, a caspase switch triggering apoptosis has shown clinical promise, allowing effective elimination of infused CAR-T cells and resolution of clinical symptoms in patients manifesting GVHD [78].

In addition to artificial receptor engineering, the functional features of T cells in the context of gene modification have also become highlighted. To achieve sufficient expansion and long-term persistence of infused gene-modified T cells following the contact with cancer cells *in vivo*, the advantages of naïve or central memory T cells for *TCR* or *CAR* gene modification have already been demonstrated [79,80]. From this viewpoint, some excellent artificial antigen-presenting cells capable of expanding gene-modified T cells retaining certain desirable phenotypes have been developed. Cell-based artificial APCs using K562 are clinically available. K562, a human erythroleukemia cell line inherently lacks almost HLA molecules which enables to minimize the allo-responsive/ non-target-specific T cell proliferation, and well tolerates gene-modification which enables to express desirable HLA class I molecule for CD8 T cell and HLA class II for CD4 T in the setting of epitope-specific T cell expansion, and to express co-stimulatory molecules including CD80, CD83, 4.1BB-L for further T cell expansion [81]. Accordingly, the use of suitable T-cell subsets for gene modification might obviate the need for chemo-radiotherapeutic lymphodepleting preconditioning, which is substantially associated with treatment-related toxicity.

Finally, the combined use of immunological checkpoint blockade, for example employing anti-cytotoxic T-lymphocyte-associated antigen (CTLA)-4 antibody [82] and anti-programmed cell death (PD)-1/PD-ligand 1 (PD-L1) antibody [83,84], which have recently yielded promising results, especially in patients with solid tumors, appears to be a promising option. In a clinical trial using anti-melanoma T cells gene-modified to express a high-avidity murine gp100-specific TCR, the infused T cells became hypofunctional for the cognate epitope via the PD-1/ PD-L1 pathway a couple of months after infusion [85], as is the case for latent retrovirus infection [86]. Similar hypofunction mediated by infused CAR-gene-modified T cells targeting mesothelin for treatment of solid tumors has also been demonstrated in a mouse model [87].

Collectively, the selection of appropriate T-cell subsets for gene modification and the blockade of immunological checkpoints should be able to improve not only the life-span, but also the functional persistence of therapeutically infused gene-modified T cells *in vivo*, resulting in better clinical outcomes.

## 6. Conlusions

In the context of antileukemia adoptive immunotherapy using T cells gene-modified to express leukemia antigen-specific receptors aimed at the cure of leukemia, the final goal is to induce durable protective immunity against disease progression in patients without collateral damage to normal tissues, and so far this has been achieved only for successful cases of allo-HSCT. For this purpose, various sophisticated approaches are currently being examined, and in the near future this treatment option might be able to take the place of allo-HSCT for patients with certain types of leukemia, especially those who are ineligible for allo-HSCT or for whom timely acquisition of a suitable donor is not possible.
